# A conversation with Jeffrey Whitsett

**DOI:** 10.1172/JCI168557

**Published:** 2023-02-15

**Authors:** Ushma S. Neill

Neonatologist and pulmonary biologist Jeffrey Whitsett (Figure 1) of the Cincinnati Children’s Hospital provided understanding of pulmonary surfactant biochemistry and structural biology that underpins the widespread application and usage of surfactant replacement therapy in neonatal respiratory distress. For the full interview, including his more recent insights into embryonic patterning in the lung, see https://www.jci.org/videos/cgms.

*JCI:* Can you tell us a little bit about your family and what you were like as a child?

Whitsett: I grew up in an Appalachian family from southern Tennessee; my grandparents on my father’s side were farmer/blacksmith types. I spent my summers chasing snakes and lizards and getting chiggers. I lived with my family in a rural small town in northern Ohio. There were only 50 or 60 kids in our local junior high school, and most didn’t go on to college. We had a bowling alley and a supermarket in our town, and that was about it. I lived outside, worked on farms, and enjoyed wildlife. I was not pressured and didn’t worry about tests. It was a real Midwest country life. My father was an artist. He ended his career in England painting portraitures and playing guitar. I grew up in a home full of art and music. I have a brother, and he’s a fine guitarist and also an emergency medicine physician.

I did fine academically without having to work very hard. I had fantastic physics, math, and chemistry teachers. Even in high school, I somehow had a fervent belief in science. I remember sitting on the porch with my father when I was seven or eight and Sputnik was passing overhead; my dad had been a pilot in World War II, and he had his own plane. We talked about it and saw this thing going across the sky, and he said, “Science is something we can use to understand life on our planet.”

*JCI:* What put you on a trajectory toward Colgate University to study chemistry?

Whitsett: I can be bought! They had a program called War Memorial Scholars that brought in those from underrepresented communities. I had a full scholarship to Colgate. I had to work serving food, but I had a full scholarship and deeply appreciated the opportunity.

*JCI:* Did you have any exposure to research during that time?

Whitsett: Since 1965, I’ve never left a research lab. It started when I was teaching natural history in a camp for children and leading hikes and the like. One of the parents of the campers was Robert Haynes, MD, PhD, a professor of pharmacology at Case Western Reserve. He invited me, even as a high school student, to work in the laboratory during college. I was always in his lab or associated labs. I just was recurrently excited and fascinated with science. It was an incredible time in science there: in the laboratory next door, Earl Sutherland and Ted Rall had discovered cAMP as a second messenger.

Bob was a fantastic mentor and scientist. He worked on mitochondrial biochemistry before it was popular. I stayed in contact with him throughout my career, but really, I started out washing their pipettes and was proud to do it. But if you dropped a 10 μl Lambda pipette in those days, that was big trouble.

*JCI:* At what point did you decide to go to medical school?

Whitsett: I was in a very small cohort pursuing chemistry, and most of us were nominally premed. The chair of the Chemistry Department, Joseph Thurner, told all the premeds that most of us would drop out of chemistry. About 12 of us didn’t, and most became physician-scientists. He was an incredible role model and a fine teacher who encouraged us to work to our full capabilities. When I graduated, he said, “Jeff, you just don’t know your limitations.” I didn’t know if that was a compliment or if he was telling me I wasn’t very bright.

*JCI:* Did you go to medical school thinking you would continue to do research?

Whitsett: Columbia University gave me a scholarship. Nixon was still president, and there were still scholarships for medicine, which was great, as my family couldn’t support me. I worked in genetics, counting chromosomes and analyzing blood for steroid levels and investigating the impact of circadian rhythms, often measuring things at three in the morning. I got my hand into research all through medical school and really enjoyed it. It was also an interesting time politically. At Columbia, the school closed down when the US bombed Cambodia. A lot of us left for a month and were active politically.

*JCI:* What drove you towards pulmonary biology and neonatology?

Whitsett: In my first year in medical school, I made rounds with Stanley James, one of the fathers of modern neonatal care. He worked with Virginia Apgar of the Apgar score, and I got to make rounds with her too. Morbidity and mortality were ever present in newborn medicine. We couldn’t take care of infants who couldn’t breathe. We couldn’t provide nutrition if they couldn’t eat. We couldn’t keep them warm. The tools for adult medicine didn’t really support babies. I knew enough about biochemistry and physiology to recognize the opportunity to bring science into this area of medicine.

In those early days, if you took meticulous care of some of these premature or other medically challenged babies, you could get some through, but they were often hopeless cases. There were huge opportunities to improve nutrition, avoid neonatal asphyxia, do blood gases, and do microanalyses to keep electrolytes in balance. All these tests couldn’t be done on tiny samples of blood until the 70s. The physiology of the 60s and understanding of cardiovascular physiology hadn’t been applied yet to babies. There was a huge opportunity to apply what we learned from internal medicine and basic science.

In my professional lifetime, neonatal mortality has gone from 30 per thousand to 5 per thousand. The babies I couldn’t take care of when I was a house staff now largely go home and live full lives. You wouldn’t believe how robust a baby is and how well they can heal and recover from real challenges.

*JCI:* Did you ever have a patient interaction during that time that solidified your path?

Whitsett: Every child we tried to ventilate in the early days died, no matter what size they were. But we had learned enough physiology, and CPAP [continuous positive airway pressure] was something just beginning to be discussed. Ventilators didn’t work because our electronics weren’t quick enough. We hyperinflated the babies, and they developed end-stage lung disease in no time at all. But CPAP was something you could build. I cared for a neurosurgeon’s child who weighed 2200 grams and now he would have less than a 1% chance of dying, but back then when I was just an intern, I knew if we put him on a ventilator, he would die. I made my own prongs with anesthesia tubing, built a bottle, and made a CPAP device. We put the prongs in the infant’s nose and held them there for three days. He went home and he’s grown up and he’s probably got a whole family himself now. I saw that being very careful and meticulous and applying fundamental ideas about physiology could be transformative. That convinced me that if we could solve this breathing problem, we could get a lot of children home.

*JCI:* Your early publications were focused on calcium signaling and placental biology.

Whitsett: I was interested in lipids and membranes from a basic biochemistry point of view. I wanted to come to Cincinnati as a clinical fellow because there was a special program in adult cardiovascular care. At the time, the apolipoproteins were being discovered and there was new insight into the transport of lipids, how membranes were maintained, and how proteins interacted with membranes. I was also interested in the fundamental question of how babies adapt to air breathing. At birth, the placenta is no longer necessary, the cord is cut, and multiple pathways must take over: metabolism, liver and cardiovascular function; the ductus arteriosa closes, and the baby must breathe — all in seconds to minutes. That transition was absolutely fascinating to me — this multiorgan biochemical and physiological transition. I started with placental studies, but saw opportunities to begin studying the lung alongside the placenta.

I read about how John Clements and his team at UCSF had shown that giving premature babies pure lipids didn’t work as an effective surfactant as a means for helping them breathe before they produced their own surfactant. The melt temperature of pure phosphatidylcholine is 41.5° to 42°. I was enough of a physical chemist to know that lipids couldn’t spread and remain stable if they were crystalline or semicrystalline at that temperature; babies’ temperatures are 36° to 37°, not 41° to 42°. I knew there had to be something in the surfactant that made the lipids move and be stable.

I proceeded with some bizarre experiments. We got free cow lungs from a meat packer in Cincinnati. We would get a whole cow lung, inflate and wash it out. We studied dozens of cow lungs and maybe 80 dogs, 3,000 rats, and many more mice and eventually purified surfactant proteins. The problem was that if you ran purified lipid extract surfactant on a gel, it was just a big smear; there were no proteins visible. The literature was not helpful and stated that proteins were not present or needed in the lipid extract. At that time, Forrest Adams [UCLA] and others had shown that an extract in surfactant works well, but it didn’t have any visible proteins. I knew there had to be proteins or some weird glycolipid that was perturbing the packing of the lipid molecules. So I put that stuff from a couple of cows into a dialysis bag and dialyzed it for a month in chloroform-methanol. Nothing after a month; I still had a big smear. But after two to three months, out came two little proteins that turned out to be SPB and SPC [surfactant proteins B and C].

In ‘82 and ‘83, we discovered these proteins that no one had found before or knew what they did. I looked at the surfactant extracts that were being used by Forest Adams, Machiko Ikegami, and Tetsuro Fujiwara and found that those two proteins were in there. We purified them, added them back to the purified lipids, and lo and behold, they made great surfactants. These were minor publications, but that’s when we discovered SPB and SPC, made antibodies against them, and got a bit of their peptide sequence. In due time, we cloned surfactant proteins A, B, C, and D, and then we knocked each of them out in mice, found the human genes, sequenced the genes, ascertained the cDNAs, and found infants with mutations in those genes. I’m still working on how they’re regulated. We later found the transcription factors that regulate their expression perinatally.

*JCI:* What was your role in the early days of surfactant replacement therapy?

Whitsett: Several companies recognized that I knew some secrets about proteins in surfactant extracts. At the time, the FDA required somewhat of a mechanistic understanding before approval: are the surfactants going to be antigenic? Are they going to be safe? Can they be manufactured? Don Shapiro, Alan Jobe, and I started working in ‘83 and ‘84 with Abbott Laboratories on a longstanding collaboration. We built on what Adams had done and what Fujiwara used in Japan and had to manufacture surfactant at scale. Manufacturing was challenging, so we worked with Abbott to make antibodies to identify the proteins to clone the genes and get a manufacturing process that would keep the proteins from precipitating and being lost. A lot of the preparations weren’t working and weren’t reliable, and we had to reengineer it for sterility reasons. We spent five years working actively to build a preparation that we could use for surfactant replacement in the US. Alan Jobe and I presented at the FDA, telling them what we were able to generate was nonantigenic, stable, didn’t cause aggregates, and didn’t misfold or cause amyloid aggregates.

The day we went to the FDA, bovine spongiform encephalopathy hit the front page. Our preparations were from cows, all from the US. So we had to reformulate and build a whole new process of getting cow lungs from New Zealand where there wasn’t any scrapie or any bovine spongiform encephalopathy.

During that time, we made many of the surfactant proteins and related peptides and tested them for surfactant replacement. The animal preparations work very well, but many investigators — including us — are working on making cheaper surfactants with small synthetic peptides.

*JCI:* If you could not have been a physician or a scientist, what kind of vocation would have kept you motivated your whole life?

Whitsett: I have a farm, 140 acres on the Kentucky River. We have two eagles bonding right now. Every year they nest, and you can see them from our front porch. I’m planting prairie grass, native perennials, and flowers where my old barn used to be. I have a few cows. I chase them when they get out of the corral and do bad things. I like physical work. I like being outside and digging ditches. I think I could have been a ditch digger and perhaps a farmer.

## Figures and Tables

**Figure 1:**
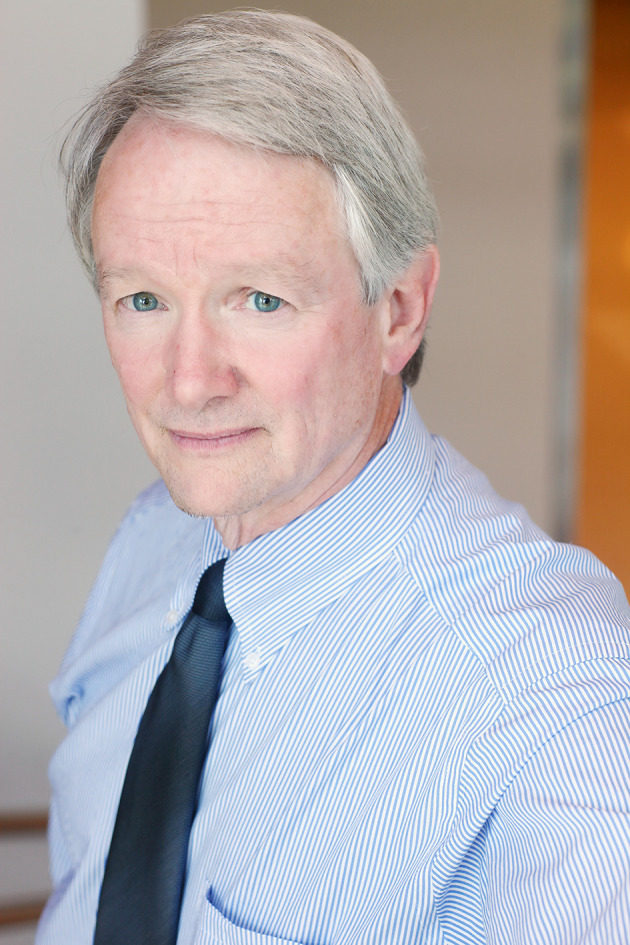
Jeffrey Whitsett.

## Version 1 02/15/2023

Electronic publication

